# All About Prostate Cancer

**DOI:** 10.1054/bjoc.2001.1783

**Published:** 2001-06

**Authors:** John R W Masters

**Affiliations:** Institute of Urology, University College London, 3rd Floor, 67 Riding House Street, London, W1W 7EY


*British Journal of Cancer* (2001) **84**(12), 1686
© 2001 Cancer Research Campaign
doi: 10.1054/ bjoc.2001.1783, available online at http://www.idealibrary.com on IDEAL®

http://www.bjcancer.com

### Book Review

### All About Prostate Cancer

Fred Stephens, Oxford University Press, 2001,
£6.99 paperback, 208 pp, 0 19 551404 1

Nowhere in the field of cancer treatment is there more controversy and confusion than is the case with prostate cancer, according to Professor Fred Stephens in his book with the grand title of 'All About Prostate Cancer'.

The prostate is the site of two of the conditions that most frequently afflict elderly men, benign prostatic hyperplasia and prostate cancer. As men age, their prostates increase in size, eventually causing irritation and obstruction of the bladder. Benign prostatic hyperplasia is not usually life-threatening, and can be managed with drugs or surgery. The same symptoms can also be caused by prostate cancer, now the most frequently diagnosed cancer in men and one of the major killers due to cancer.

Prostate cancer is lethal, and yet most men die with, rather than of, the disease. If a man lives long enough, he will almost certainly have microscopic evidence of prostate cancer at autopsy. Prostate cancer tends to grow slowly and present late in life and it is these two characteristics that lead to the controversy and confusion over treatment.

Fred Stephens took up his pen after retiring as Head of Surgery at the University of Sydney Medical School in Australia. He describes the choices given to men faced with a diagnosis of prostate cancer. For early disease, still localized within the prostate, difficult decisions must be made. For cancer that has spread beyond the prostate, there is less debate and almost the only course of action that will extend survival is castration, medical or surgical.

Almost everyone in the cancer business has stressed the need for early diagnosis and treatment as the best, and almost the only, opportunity for an individual to beat his or her cancer. Yet in prostate cancer we are unsure about the value of screening and early diagnosis. Perhaps this is less surprising when we accept that we do not always know whether it is worth treating the disease, even when it has been diagnosed. For many patients, and particularly those over 70 with about 10 years or so left of their lives, a 'watch and wait' policy is preferred. Fred Stephens gently rambles around these issues, without being prescriptive or taking sides, and repeats the more important points many times.

So why would a man want to leave a cancer inside him, letting it grow and perhaps metastasize, spurning the brief window of opportunity of being cured. There is no short or easy answer to this question – the issues are complex and debated with great intensity. First of all, there is life expectancy. If you are a man over 70 years old with perhaps another 10 years to live, do you want a therapy with many unpleasant side-effects for a disease that is unlikely to kill you before you die of something else? If you are less than 70 years old, you will probably opt for one of two treatments, radical prostatectomy or radical radiotherapy, and accept the variable degree of impotence and incontinence that will result.

Radical therapy has the potential to cure prostate cancer. But medical opinion can change rapidly. Only a decade ago it was said that cure was not necessary in many of the men in whom it was possible and cure was no longer possible in many of the men in whom it was needed. Is this still true? It is a fact that staging procedures are of limited accuracy. Consequently 30–40% of men given radical treatment have disease that extends beyond the prostate. The other side of the coin is that some of the men who are cured did not need the treatment, at least to the extent that the disease would not have caused them medical problems within their lifetime.

Prostate cancer has developed a high profile and this book adds an Australian dimension to an avalanche of books, pamphlets and information on the world wide web. Fred Stephens shares his beliefs, his attitudes and what he considers to be the best treatment with us. He does not tell us why he wrote the book or whom it is for. There is an implication that the book is for a lay audience, but he often seems to be addressing fellow professionals, and consequently misses the target for both audiences.

*John RW Masters*
*Professor of Experimental Pathology*
*Institute of Urology*
*University College London*
*3rd Floor*
*67 Riding House Street*
*London W1W 7EY*

